# Comprehensive immune transcriptomic analysis in bladder cancer reveals subtype specific immune gene expression patterns of prognostic relevance

**DOI:** 10.18632/oncotarget.20237

**Published:** 2017-08-09

**Authors:** Runhan Ren, Kathrin Tyryshkin, Charles H. Graham, Madhuri Koti, D. Robert Siemens

**Affiliations:** ^1^ Department of Urology, Queen’s University, Kingston, ON, Canada; ^2^ Department of Biomedical and Molecular Sciences, Queen’s University, Kingston, ON, Canada; ^3^ Department of Pathology and Molecular Medicine, Queen’s University, Kingston, ON, Canada; ^4^ Cancer Biology and Genetics Division, Queen’s Cancer Research Institute, Queen’s University, Kingston, ON, Canada; ^5^ Department of Obstetrics and Gynecology, Queen’s University, Kingston, ON, Canada

**Keywords:** MIBC, immune biomarkers, immunotherapy, TCGA, interferon

## Abstract

Recent efforts on genome wide profiling of muscle invasive bladder cancer (MIBC) have led to its classification into distinct genomic and transcriptomic molecular subtypes that exhibit variability in prognosis. Evolving evidence from recent immunotherapy trials has demonstrated the significance of pre-existing tumour immune profiles that could guide treatment decisions. To identify immune gene expression patterns associated with the molecular subtypes, we performed a comprehensive *in silico* immune transcriptomic profiling, utilizing transcriptomic data from 347 MIBC cases from The Cancer Genome Atlas (TCGA). To investigate subtype-associated immune gene expression patterns, we assembled 924 immune response genes and specifically those involved in T-cell cytotoxicity and the Type I/II interferon pathways. A set of 157 ranked genes was able to distinguish the four subtypes in an unsupervised analysis in an original training cohort (n=122) and an expanded, validation cohort (n=225). The most common overrepresented pathways distinguishing the four molecular subtypes, included JAK/STAT signaling, Toll-like receptor signaling, interleukin signaling, and T-cell activation. Some of the most enriched biological processes were responses to IFN-γ, antigen processing and presentation, cytokine mediated signaling, hemopoeisis, cell proliferation and cellular defense response in the TCGA cluster IV. Our novel findings provide further insights into the association between genomic subtypes and immune activation in MIBC and may open novel opportunities for their exploitation towards precise treatment with immunotherapy.

## INTRODUCTION

Urothelial bladder cancer (UBC) is the fifth most common cancer worldwide [1] and is one of the most management intensive cancers in North America [2]. Although the majority of incident cases of UBC are non-invasive at presentation, muscle invasive bladder cancer (MIBC) represents very aggressive disease with rapid progression to metastases [3] and poor overall survival despite intensive local and systemic therapy. Current standards for localized MIBC include radical cystectomy with or without perioperative cisplatin-based chemotherapy [3]. Unfortunately, many suffer early disease recurrence and, despite palliative chemotherapy, median survival rates are generally less than one year [4]. The optimal management of patients with higher risk UBC is ambiguous with a significant need for better prediction tools and enhanced therapeutics [5].

MIBCs are highly heterogeneous tumours. Recent investigations based on molecular profiling of specimens from large UBC cohorts have led to their classification into molecular subtypes that display distinct genomic and transcriptomic features, resembling those seen in breast cancer [3], [6–8]. Interestingly, these subtypes may exhibit distinct associations with treatment response and survival [8, 9]. Although different groups have classified UBC into two [8], three [3], four [6], or five [7] subtypes, there is a consensus that the top separation occurs as the basal and luminal subtypes [10]. Basal tumours, enriched with EGFR and hypoxia-inducible factor 1 expression, are often metastatic at presentation, possess squamous and sarcomatoid histological features, and have epithelial-to-mesenchymal transition cell biomarkers [11]. In comparison, luminal cancers have papillary features and commonly *FGFR3*, *ERBB2*, and *ERBB3* activating mutations [11]. The Cancer Genome Atlas Network (TCGA) bladder analysis working group classified bladder tumours into four clusters named I, II, III, and IV [6]. Clusters I and II correspond to the luminal subtype, while III and IV represent the basal subtype [12]. Tumours in Cluster I are enriched in *FGFR3* overexpression due to mutations and amplification and show better overall survival, whereas those in cluster II, designated “p53-like” tumours, express active p53 gene signatures and are resistant to neoadjuvant cisplatin-based combination chemotherapy [3]. Cluster IV shares features with the claudin-low subtype of breast cancer, express immune checkpoint molecules, and were actively immunosuppressed, despite having an enriched immune gene signature [13]. In particular, Kardos et al. [13] demonstrated that immune infiltration was not correlated with predicted neoantigen burden, but from unopposed NF-kB activity from downregulated PPARγ signaling.

Given the urgent need of alternative approaches in MIBC treatment, there has been a growing interest in immunotherapies, such as those targeting the immune checkpoints: CTLA-4, PD-L1, and PD-1 [14]. Atezolizumab, a PD-L1 inhibitor, has been recently approved by the FDA for bladder cancer that progressed during or following chemotherapy [15]. Evolving evidence based on the success of immune checkpoint blockade therapies in melanoma and non-small cell lung cancer has confirmed the significance of the pre-treatment tumour immune state as a strong prognostic and response predictive indicator [14, 16]. An important feature, key to the success of immunotherapy, is the spatial organization of cytotoxic CD8^+^ tumour infiltrating lymphocytes (TILs) in the epithelial and stromal compartments and their activation status [17]. Higher density of CD3^+^ and CD8^+^ TILs have been associated with increased disease-free and overall survival in melanoma, head and neck, breast, bladder, urothelial, ovarian, colorectal, prostatic, and lung cancer; however, their activation status determines their prognostic significance in most cancers [18, 19]. In particular the expression of interferons (IFNs), which play a central role in anti-tumour immune responses, are emerging as prognostic and predictive biomarkers of both chemotherapy and immunotherapy [20]. Higher infiltration with CD4^+^ and regulatory subsets of TILs and higher CD68 to CD3 ratios are associated with poor prognosis in bladder cancer [21–23]. In particular PD-L1, IDO, FOXP3, TIM3, and LAG3 are expressed in T-cell-inflamed, and β-catenin, PPAR-γ, and FGFR3 in non-T-cell-inflamed urothelial tumours [17]. Although the pre-treatment expression of PD-1/PD-L1 initially showed some predictive value, it has recently failed to perform as a good biomarker in the recent clinical trials due to their transient nature of expression [23–25].

As reported in other cancers sites, it is likely that the pre-existing tumour immune landscape in UBC could be an additive determinant of response to chemotherapy as well as immune-based therapies leading to more precise prognostication, patient stratification, and informed treatment decisions [26]. To our knowledge there are no previous studies in MIBC that have evaluated the association between immune transcriptomic alterations, specifically those mediated by IFNs and cytotoxic pathway genes, and their potential associations with their distinct molecular sub-populations. In the current study, we performed a comprehensive *in silico* immune transcriptomic profiling of MIBC using the publicly available TCGA global transcriptomic datasets in order to determine whether the known molecular subtypes of MIBC are associated with specific immune gene signatures. The findings from our study may not only provide insights into the association between genomic subtypes and immune activation, but may also open novel opportunities for improving the management of MIBC.

## RESULTS

We aimed to determine whether the previously defined four TCGA MIBC clusters exhibit differences in their immune gene expression patterns that could be of potential significance in informing treatment decisions for immunotherapies and other combinatorial treatment approaches.

### Clinicopathological features of TCGA MIBC cohort

The TCGA cohort as reported earlier, consisted of chemotherapy-naïve, muscle-invasive, high-grade urothelial tumors (T2-T4a, Nx, Mx) [6]. Inclusion criteria reviewed by five expert genitourinary pathologists involved: tumour nuclei ≥ 60% of total, ≤20% tumour necrosis in the specimen, and variant histology ≤50% [6].

### Immune gene expression patterns across MIBC clusters

**Figure 1:**
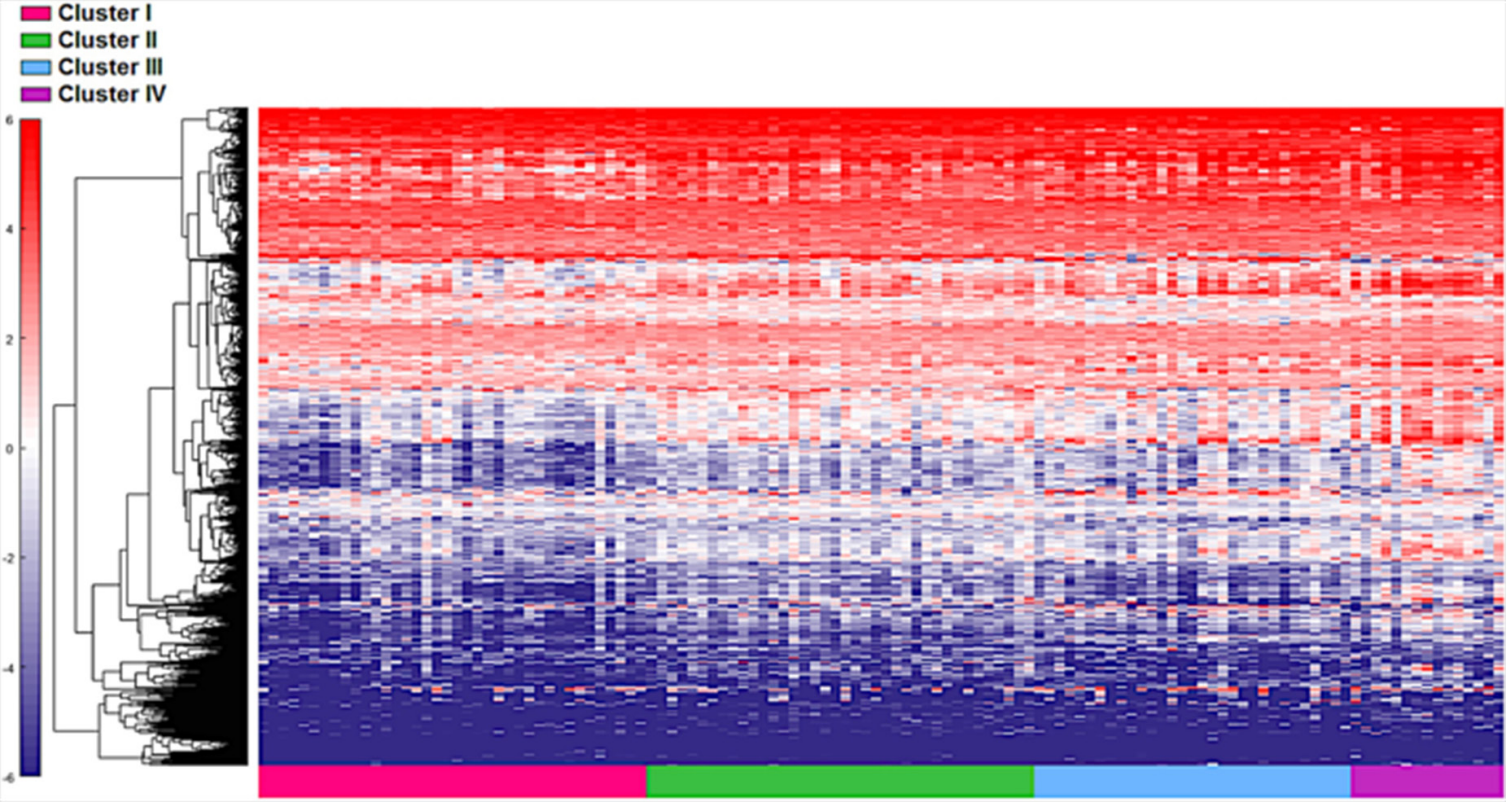
Distinct immune gene expression levels in cohort 1 (n=122) between the four TCGA bladder cancer subtypes based on the top 20% (377 NanosString panel genes) using the feature selection algorithm. Red indicates high expression, and blue indicates low expression.

**Figure 2:**
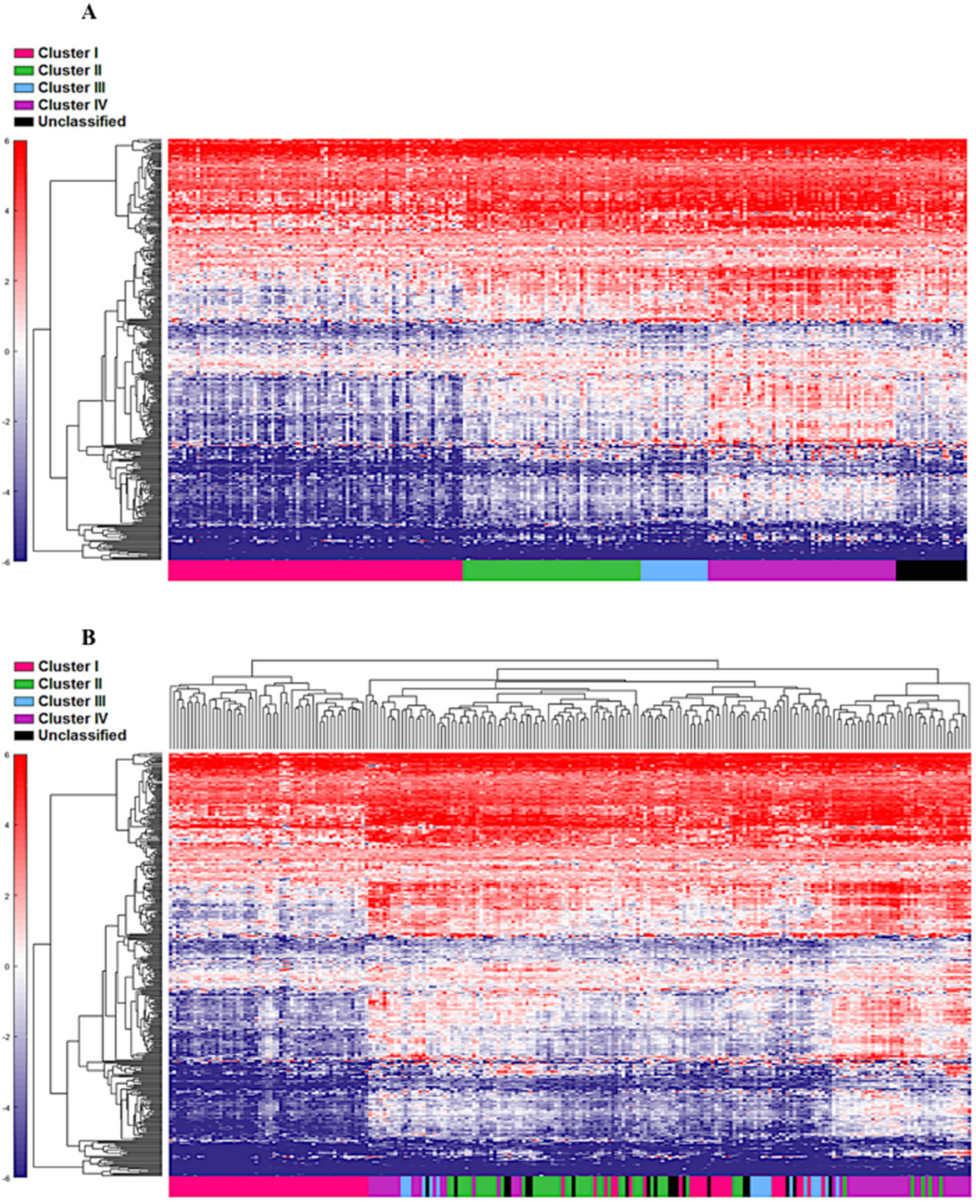
Cohort 2 (n=225) assigned to clusters based on Euclidian distance to the cluster centroids generated from the cohort 1 (n=122) Supervised **(A)** and unsupervised **(B)** analysis based on the samples and 377 NanoString™ panel genes.

**Figure 3:**
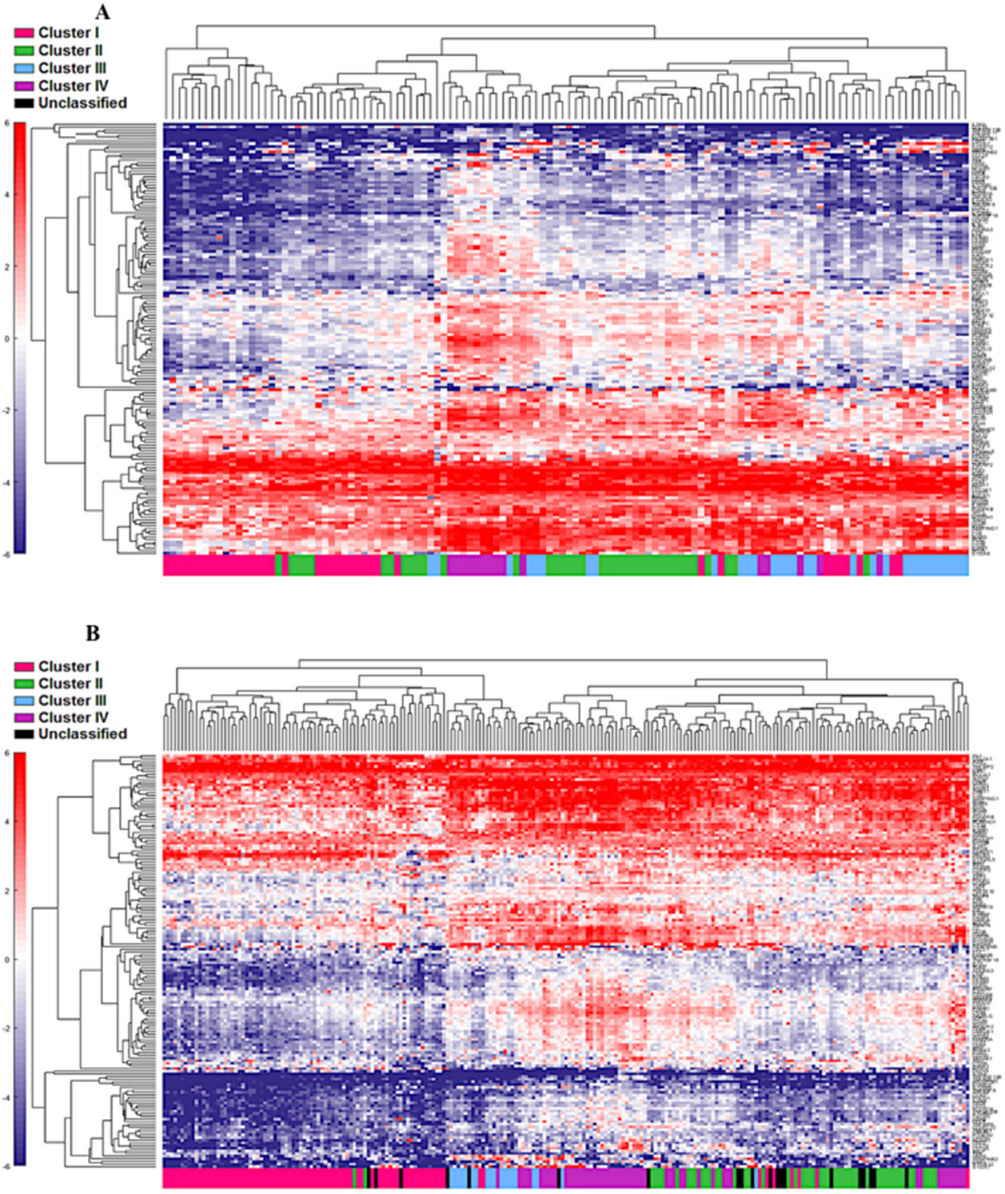
Unsupervised analysis of both the cohort 1 **(A)** and cohort 2 **(B)** using the top 5% (n=157) genes. Unsupervised grouping shows gradient of under expression in cluster I to overexpression in cluster IV.

First, the 122 samples previously used to identify the four clusters by TCGA [6] were treated as a discovery group to determine immune gene expression profiles across clusters. A total of 377 genes derived from the NanoString™ panel discriminating among the clusters were identified using a feature selection technique (Supplementary Table 1). The performance of these genes to accurately distinguish the four TCGA clusters was evaluated by clustering of cohort 1 (Figure 1). This set of genes was then used to assign samples in the validation set to the four clusters. Similar to cohort 1, the genes were able to distinguish the four clusters in cohort 2 by supervised and unsupervised analysis (Figure 2A and 2B). Similar unsupervised analysis was done using the top 5% of genes derived from all immune panels (n=157) (Supplementary Table 2) on both cohorts (Figure 3A and 3B). A recent updated analysis of the current TCGA bladder tumour cohort shows that clusters I-IV remained stable [28], supporting our classification approach in cohorts 1 and 2.

### Differential pre-existing expression patterns of interferon associated genes

**Figure 4:**
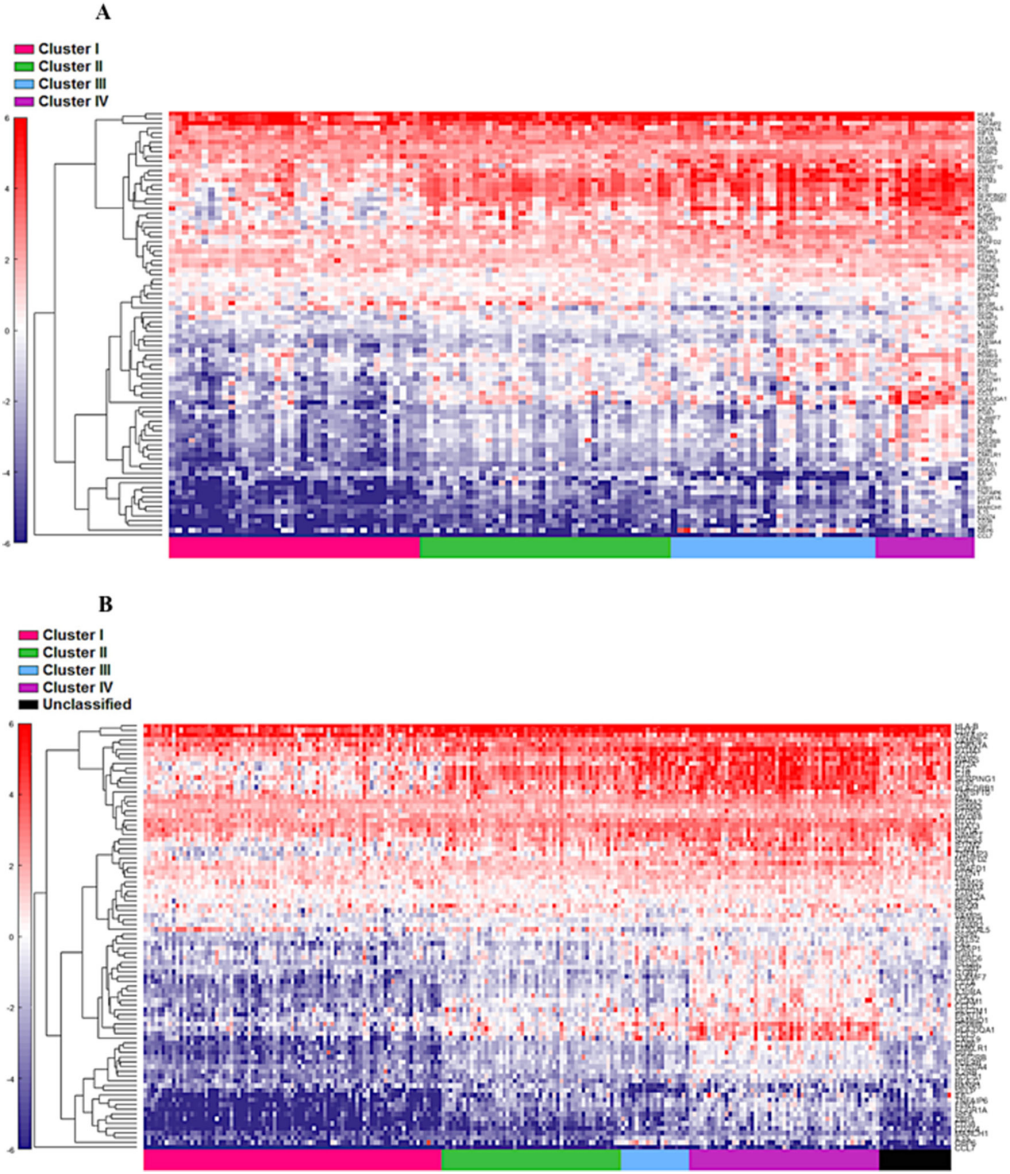
Supervised heat map of top 20% of IFN-γ associated pathway genes in both cohort 1 **(A)** and cohort 2 **(B).**

**Figure 5:**
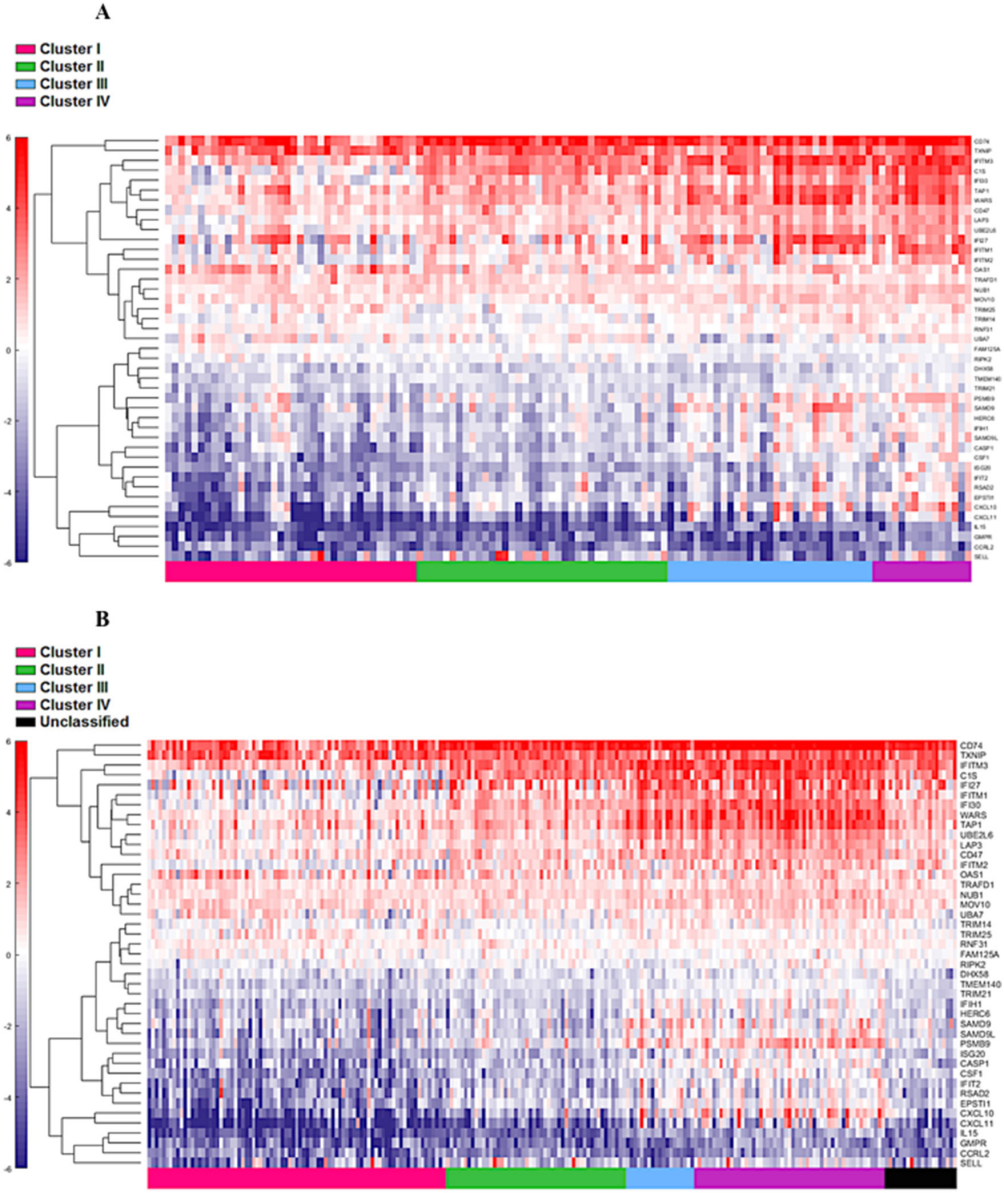
Supervised heat map of top 20% of IFN-α associated pathway genes in both discovery **(A)** and validation **(B)** groups.

**Figure 6:**
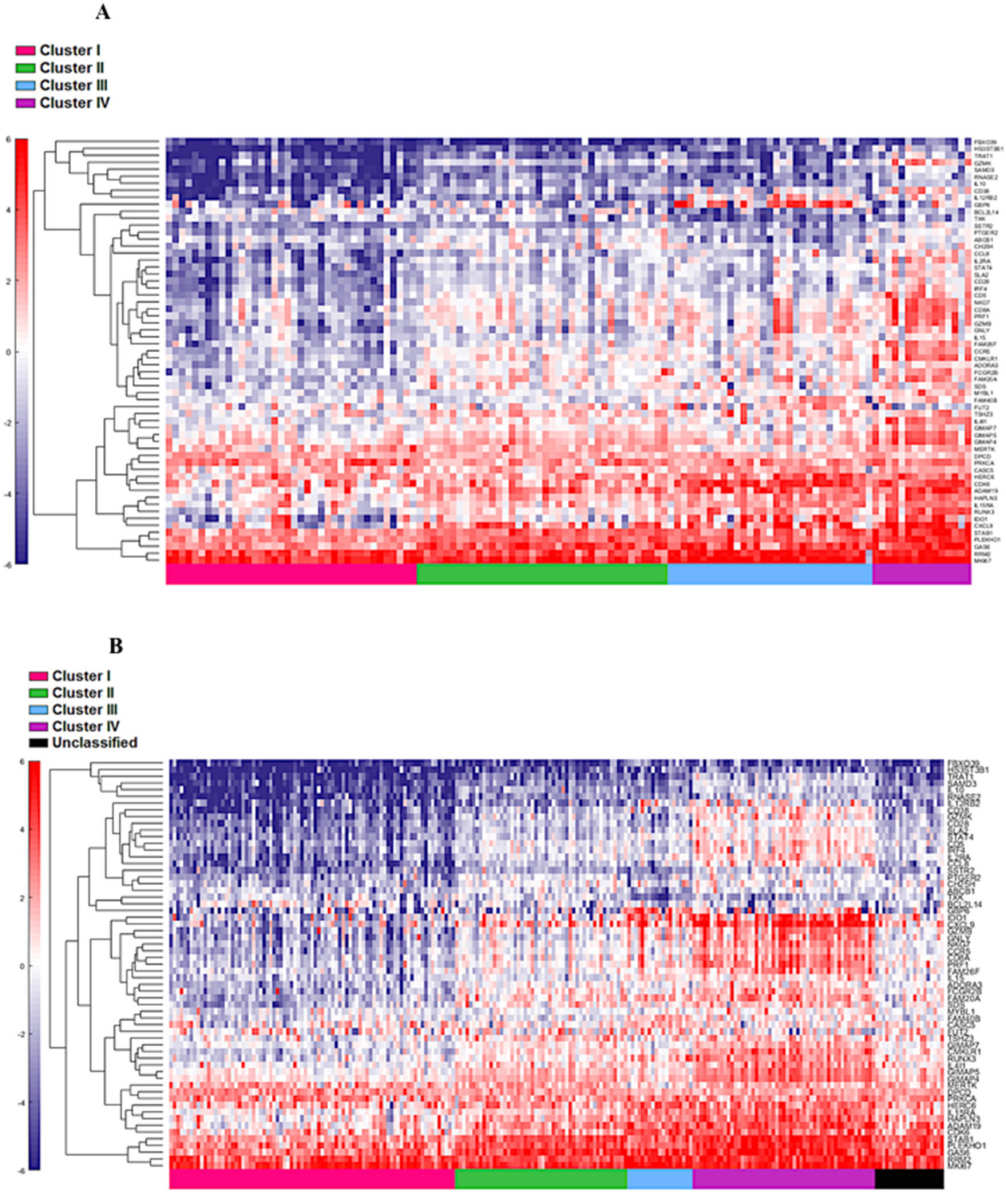
Supervised heat map of top 20% of cytotoxic associated pathway genes in both cohort 1 **(A)** and cohort 2 **(B).**

The four cluster patterns were also noted for the top 20% ranking IFN-γ associated genes upon hierarchical clustering analysis in both training and validation cohorts (Figure 4A and 4B). A gradient of under-expression of IFN-γ associated genes in cluster I to overexpression in cluster IV is observed in both. A similar pattern was also noted in the top 20% ranking IFN-α (Figure 5A and 5B) and cytotoxic genes (Figure 6A and 6B). Most importantly, key IFN response genes and downstream T-cell recruiting target chemokine genes*, CXCL9*, *CXCL10*, and *CXCL11*, and their common receptor *CXCR3*, showed increased expression in clusters III and IV. Similarly, others in the list included the key players in IFN response such as *IFITM2, CCL5, IRF4* and others.

### Antigen processing pathways are overrepresented in T-cell inflamed MIBC clusters

**Figure 7:**
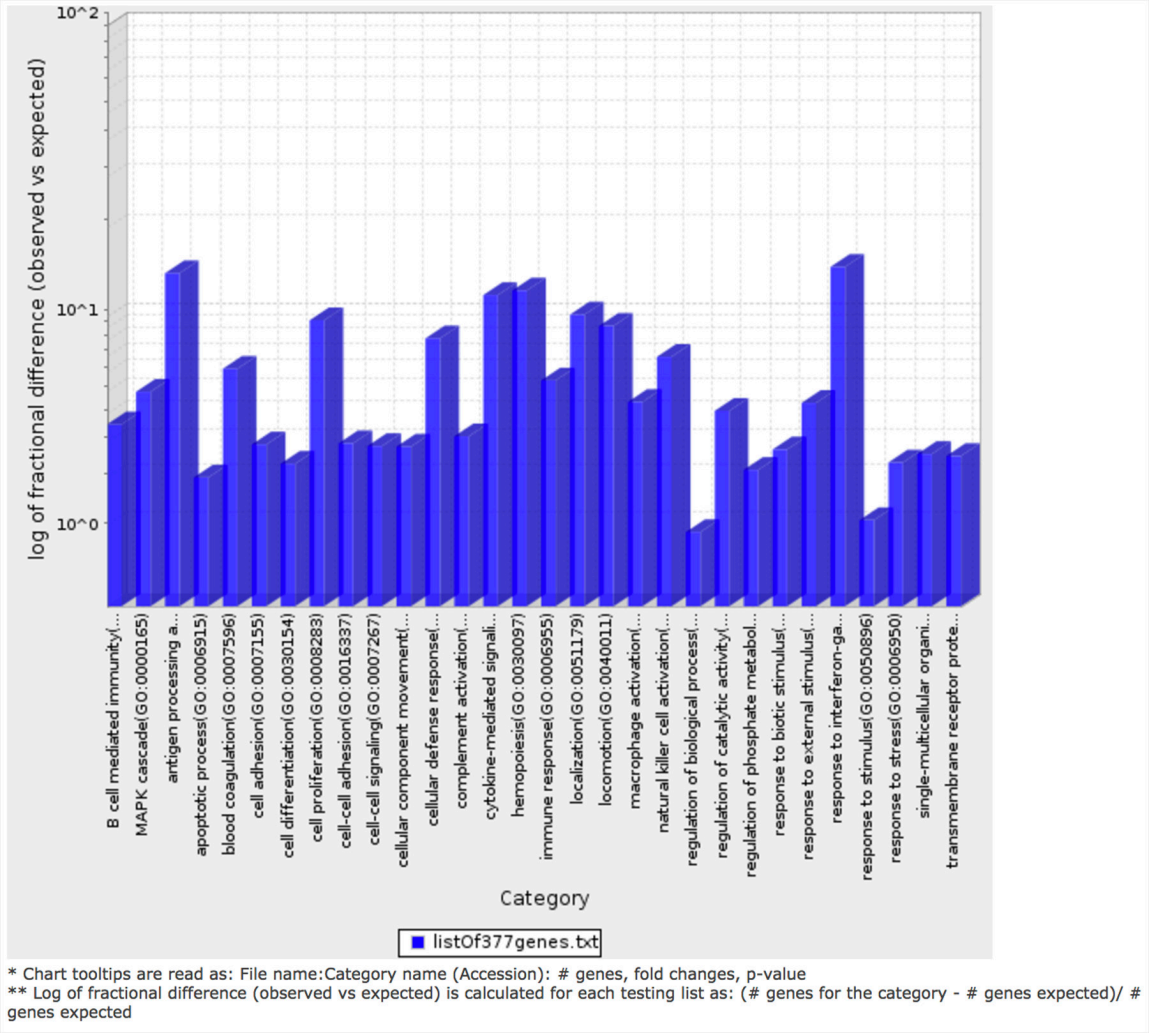
Bar graph depicting distribution of fold enrichment levels of biological pathways defined by PANTHER based analysis in the 377 genes that show differential expression patterns in the four TCGA MIBC clusters The enriched categories were obtained upon analysis using the statistical overrepresentation test defined by PANTHER tool [27].

We determined the Gene Ontology functional annotations of the differentially expressed genes that distinguished the four clusters, using both the 377 and 157 genes as input gene lists. Using the overrepresentation statistic in PANTHER, we calculated the probability of highly populated protein classes and gene ontology classes among the two gene lists (Table 2a and 2b). The most enriched GO biological processes in the 377-gene list were response to IFN-γ, antigen processing and presentation, cytokine mediated signaling, hemopoeisis, cell proliferation and cellular defense response (Supplementary Table 3). The top five overrepresented pathways included JAK/STAT signaling pathway, Toll-like receptor signaling pathway, interleukin signalling pathway, and T-cell activation (Figure 7). Interestingly, similar analysis using the top ranking 157 genes as input list, revealed only the B-cell activation, T-cell activation, and inflammation mediated by chemokine and cytokine signaling pathways as the only three overrepresented pathways. Response to IFN-γ, hemopoiesis, macrophage activation and cell proliferation were the most overrepresented GO biological processes in the top ranked 157 genes (Supplementary Table 4).

**Table 1:** Custom designed immune gene panel of 924 genes, consisting of IFN-α and IFN-γ pathway genes from GSEA and immune response genes defined by the NanoString nCounter PanCancer immune Pathways Panel

Immune gene panel - NanoString nCounter PanCancer immune panel				
A2M	C1R	CCND3	CD44	CLEC5A
ABCB1	C1S	CCR1	CD46	CLEC6A
ABL1	C2	CCR2	CD47	CLEC7A
ADA	C3	CCR3	CD48	CLU
ADORA2A	C3AR1	CCR4	CD5	CMA1
AICDA	C4BPA	CCR5	CD53	CMKLR1
AIRE	C5	CCR6	CD55	COL3A1
AKT3	C6	CCR7	CD58	COLEC12
ALCAM	C7	CCR9	CD59	CR1
AMBP	C8A	CCRL2	CD6	CR2
AMICA1	C8B	CD14	CD63	CREB1
ANP32B	C8G	CD160	CD68	CREB5
ANXA1	C9	CD163	CD7	CREBBP
APOE	CAMP	CD164	CD70	CRP
APP	CARD11	CD180	CD74	CSF1
ARG1	CARD9	CD19	CD79A	CSF1R
ARG2	CASP1	CD1A	CD79B	CSF2
ATF1	CASP10	CD1B	CD80	CSF2RB
ATF2	CASP3	CD1C	CD81	CSF3
ATG10	CASP8	CD1D	CD83	CSF3R
ATG12	CCL1	CD1E	CD84	CT45A1
ATG16L1	CCL11	CD2	CD86	CTAG1B
ATG5	CCL13	CD200	CD8A	CTAGE1
ATG7	CCL14	CD207	CD8B	CTCFL
ATM	CCL15	CD209	CD9	CTLA4
AXL	CCL16	CD22	CD96	CTSG
BAGE	CCL17	CD24	CD97	CTSH
BATF	CCL18	CD244	CD99	CTSL1
BAX	CCL19	CD247	CDH1	CTSS
BCL10	CCL2	CD27	CDH5	CTSW
BCL2	CCL20	CD274	CDK1	CX3CL1
BCL2L1	CCL21	CD276	CDKN1A	CX3CR1
BCL6	CCL22	CD28	CEACAM1	CXCL1
BID	CCL23	CD33	CEACAM6	CXCL10
BIRC5	CCL24	CD34	CEACAM8	CXCL11
BLK	CCL25	CD36	CEBPB	CXCL12
BLNK	CCL26	CD37	CFB	CXCL13
BMI1	CCL27	CD38	CFD	CXCL14
BST1	CCL28	CD3D	CFI	CXCL16
BST2	CCL3	CD3E	CFP	CXCL2
BTK	CCL3L1	CD3EAP	CHIT1	CXCL3
BTLA	CCL4	CD3G	CHUK	CXCL5
C1QA	CCL5	CD4	CKLF	CXCL6
C1QB	CCL7	CD40	CLEC4A	CXCL9
C1QBP	CCL8	CD40LG	CLEC4C	CXCR1
CXCR2	FOS	IFI27	IL19	IRAK4
CXCR3	FOXJ1	IFI35	IL1A	IRF1
CXCR4	FOXP3	IFIH1	IL1B	IRF2
CXCR5	FPR2	IFIT1	IL1R1	IRF3
CXCR6	FUT5	IFIT2	IL1R2	IRF4
CYBB	FUT7	IFITM1	IL1RAP	IRF5
CYFIP2	FYN	IFITM2	IL1RAPL2	IRF7
CYLD	GAGE1	IFNA1	IL1RL1	IRF8
DDX43	GATA3	IFNA17	IL1RL2	IRGM
DDX58	GNLY	IFNA2	IL1RN	ISG15
DEFB1	GPI	IFNA7	IL2	ISG20
DMBT1	GPR44	IFNA8	IL21	ITCH
DOCK9	GTF3C1	IFNAR1	IL21R	ITGA1
DPP4	GZMA	IFNAR2	IL22	ITGA2
DUSP4	GZMB	IFNB1	IL22RA1	ITGA2B
DUSP6	GZMH	IFNG	IL22RA2	ITGA4
EBI3	GZMK	IFNGR1	IL23A	ITGA5
ECSIT	GZMM	IGF1R	IL23R	ITGA6
EGR1	HAMP	IGF2R	IL24	ITGAE
EGR2	HAVCR2	IGLL1	IL25	ITGAL
ELANE	HCK	IKBKB	IL26	ITGAM
ELK1	HLA-A	IKBKE	IL27	ITGAX
ENG	HLA-B	IKBKG	IL28A	ITGB1
ENTPD1	HLA-C	IL10	IL29	ITGB2
EOMES	HLA-DMA	IL10RA	IL2RA	ITGB3
EP300	HLA-DMB	IL11	IL2RB	ITGB4
EPCAM	HLA-DOB	IL11RA	IL2RG	ITK
ETS1	HLA-DPA1	IL12A	IL3	JAK1
EWSR1	HLA-DPB1	IL12B	IL32	JAK2
F12	HLA-DQA1	IL12RB1	IL34	JAK3
F13A1	HLA-DQB1	IL12RB2	IL3RA	JAM3
F2RL1	HLA-DRA	IL13	IL4	KIR2DL1
FADD	HLA-E	IL13RA1	IL4R	KIR2DL3
FAS	HLA-G	IL13RA2	IL5	KIR3DL1
FCER1A	HMGB1	IL15	IL5RA	KIR3DL2
FCER1G	HRAS	IL15RA	IL6	KIR3DL3
FCER2	HSD11B1	IL16	IL6R	KIT
FCGR1A	ICAM1	IL17A	IL6ST	KLRB1
FCGR2A	ICAM2	IL17B	IL7	KLRC1
FCGR2B	ICAM3	IL17F	IL7R	KLRC2
FCGR3A	ICAM4	IL17RA	IL9	KLRD1
FEZ1	ICOS	IL17RB	ILF3	KLRF1
FLT3	ICOSLG	IL18	INPP5D	KLRG1
FLT3LG	IDO1	IL18R1	IRAK1	KLRK1
FN1	IFI16	IL18RAP	IRAK2	LAG3
LAIR2	MAPK3	NT5E	RELB	STAT2
LAMP1	MAPK8	NUP107	REPS1	STAT3
LAMP2	MAPKAPK2	OAS3	RIPK2	STAT4
LAMP3	MARCO	OSM	ROPN1	STAT5B
LBP	MASP1	PASD1	RORA	STAT6
LCK	MASP2	PAX5	RORC	SYCP1
LCN2	MAVS	PBK	RPS6	SYK
LCP1	MBL2	PDCD1	RRAD	SYT17
LGALS3	MCAM	PDCD1LG2	RUNX1	TAB1
LIF	MEF2C	PDGFC	RUNX3	TAL1
LILRA1	MEFV	PDGFRB	S100A12	TANK
LILRA4	MERTK	PECAM1	S100A7	TAP1
LILRA5	MFGE8	PIK3CD	S100A8	TAP2
LILRB1	MICA	PIK3CG	S100B	TAPBP
LILRB2	MICB	PIN1	SAA1	TARP
LILRB3	MIF	PLA2G1B	SBNO2	TBK1
LRP1	MME	PLA2G6	SELE	TBX21
LRRN3	MNX1	PLAU	SELL	TCF7
LTA	MPPED1	PLAUR	SELPLG	TFE3
LTB	MR1	PMCH	SEMG1	TFEB
LTBR	MRC1	PNMA1	SERPINB2	TFRC
LTF	MS4A1	POU2AF1	SERPING1	TGFB1
LTK	MS4A2	POU2F2	SH2B2	TGFB2
LY86	MSR1	PPARG	SH2D1A	THBD
LY9	MST1R	PPBP	SH2D1B	THBS1
LY96	MUC1	PRAME	SIGIRR	THY1
LYN	MX1	PRF1	SIGLEC1	TICAM1
MAF	MYD88	PRG2	SLAMF1	TICAM2
MAGEA1	NCAM1	PRKCD	SLAMF6	TIGIT
MAGEA12	NCF4	PRKCE	SLAMF7	TIRAP
MAGEA3	NCR1	PRM1	SLC11A1	TLR1
MAGEA4	NEFL	PSEN1	SMAD2	TLR10
MAGEB2	NFATC1	PSEN2	SMAD3	TLR2
MAGEC1	NFATC2	PSMB10	SMPD3	TLR3
MAGEC2	NFATC3	PSMB7	SOCS1	TLR4
MAP2K1	NFATC4	PSMB8	SPA17	TLR5
MAP2K2	NFKB1	PSMB9	SPACA3	TLR6
MAP2K4	NFKB2	PSMD7	SPINK5	TLR7
MAP3K1	NFKBIA	PTGS2	SPN	TLR8
MAP3K5	NLRC5	PTPRC	SPO11	TLR9
MAP3K7	NLRP3	PVR	SPP1	TMEFF2
MAP4K2	NOD1	PYCARD	SSX1	TNF
MAPK1	NOD2	RAG1	SSX4	TNFAIP3
MAPK11	NOTCH1	REL	ST6GAL1	TNFRSF10B
MAPK14	NRP1	RELA	STAT1	TNFRSF10C
TNFRSF11A	TNFRSF4	TNFSF18	TREM1	VEGFA
TNFRSF11B	TNFRSF8	TNFSF4	TREM2	VEGFC
TNFRSF12A	TNFRSF9	TNFSF8	TTK	XCL2
TNFRSF13B	TNFSF10	TOLLIP	TXK	XCR1
TNFRSF13C	TNFSF11	TP53	TXNIP	YTHDF2
TNFRSF14	TNFSF12	TPSAB1	TYK2	ZAP70
TNFRSF17	TNFSF13	TPTE	UBC	ZNF205
TNFRSF18	TNFSF13B	TRAF2	ULBP2	
TNFRSF1A	TNFSF14	TRAF3	USP9Y	
TNFRSF1B	TNFSF15	TRAF6	VCAM1	

Immune gene panel – IFN-γ associated genes				
ADAR	CSF2RB	IFI44	LGALS3BP	PNP
APOL6	CXCL10	IFI44L	LY6E	PNPT1
ARID5B	CXCL11	IFIH1	LYSMD2	PRIC285
ARL4A	CXCL9	IFIT1	MAR-01	PSMA2
AUTS2	DDX58	IFIT2	METTL7B	PSMA3
B2M	DDX60	IFIT3	MT2A	PSMB10
BANK1	DHX58	IFITM2	MTHFD2	PSMB2
BATF2	EIF2AK2	IFITM3	MVP	PSMB8
BPGM	EIF4E3	IFNAR2	MX1	PSMB9
BST2	EPSTI1	IL10RA	MX2	PSME1
BTG1	FAS	IL15	MYD88	PSME2
C1R	FCGR1A	IL15RA	NAMPT	PTGS2
C1S	FGL2	IL18BP	NCOA3	PTPN1
CASP1	FPR1	IL2RB	NFKB1	PTPN2
CASP3	FTSJD2	IL4R	NFKBIA	PTPN6
CASP4	GBP4	IL6	NLRC5	RAPGEF6
CASP7	GBP6	IL7	NMI	RBCK1
CASP8	GCH1	IRF1	NOD1	RIPK1
CCL2	GPR18	IRF2	NUP93	RIPK2
CCL5	GZMA	IRF4	OAS2	RNF213
CCL7	HERC6	IRF5	OAS3	RNF31
CD274	HIF1A	IRF7	OASL	RSAD2
CD38	HLA-A	IRF8	OGFR	RTP4
CD40	HLA-B	IRF9	P2RY14	SAMD9L
CD69	HLA-DMA	ISG15	PARP12	SAMHD1
CD74	HLA-DQA1	ISG20	PARP14	SECTM1
CD86	HLA-DRB1	ISOC1	PDE4B	SELP
CDKN1A	HLA-G	ITGB7	PELI1	SERPING1
CFB	ICAM1	JAK2	PFKP	SLAMF7
CFH	IDO1	KLRK1	PIM1	SLC25A28
CIITA	IFI27	LAP3	PLA2G4A	SOCS1
CMKLR1	IFI30	LATS2	PLSCR1	SOCS3
CMPK2	IFI35	LCP2	PML	SOD2
SP110	STAT2	TNFAIP3	TRIM25	VAMP8
SPPL2A	STAT3	TNFAIP6	TRIM26	VCAM1
SRI	STAT4	TNFSF10	TXNIP	WARS
SSPN	TAP1	TOR1B	UBE2L6	XAF1
ST3GAL5	TAPBP	TRAFD1	UPP1	XCL1
ST8SIA4	TDRD7	TRIM14	USP18	ZBP1
STAT1	TNFAIP2	TRIM21	VAMP5	ZNFX1

Immune gene panel – IFN-α associated genes				
ADAR	FAM125A	IL4R	OGFR	SLC25A28
B2M	FAM46A	IL7	PARP12	SP110
BATF2	FTSJD2	IRF1	PARP14	STAT2
BST2	GBP2	IRF2	PARP9	TAP1
C1S	GBP4	IRF7	PLSCR1	TDRD7
CASP1	GMPR	IRF9	PNPT1	TMEM140
CASP8	HERC6	ISG15	PRIC285	TRAFD1
CCRL2	HLA-C	ISG20	PROCR	TRIM14
CD47	IFI27	LAMP3	PSMA3	TRIM21
CD74	IFI30	LAP3	PSMB8	TRIM25
CMPK2	IFI35	LGALS3BP	PSMB9	TRIM26
CNP	IFI44	LPAR6	PSME1	TRIM5
CSF1	IFI44L	LY6E	PSME2	TXNIP
CXCL10	IFIH1	MOV10	RIPK2	UBA7
CXCL11	IFIT2	MX1	RNF31	UBE2L6
DDX60	IFIT3	NCOA7	RSAD2	USP18
DHX58	IFITM1	NMI	RTP4	WARS
EIF2AK2	IFITM2	NUB1	SAMD9	
ELF1	IFITM3	OAS1	SAMD9L	
EPSTI1	IL15	OASL	SELL	

Immune gene panel – cytotoxic associated genes				
ABCB1	CCL8	CTLA4	FFAR3	HERC6
ADAM19	CCR5	CXCL10	FUT2	HESX1
ADAM3A	CD163L1	CXCL11	GAS6	HS3ST3B1
ADORA3	CD28	CXCL9	GBP6	IDO1
ANKRD22	CD300E	DNA2	GIMAP4	IFNB1
APOL3	CD38	DPCD	GIMAP5	IFNG
ARNT2	CD5	ENPP2	GIMAP7	IL10
ATF5	CD8A	F13A1	GIMAP8	IL12RB2
BATF2	CDC7	F2R	GNLY	IL15
BCL2	CDK6	FAM20A	GPR171	IL15RA
BCL2L14	CH25H	FAM26F	GZMB	IL21
C5orf39	CMKLR1	FAM40B	GZMK	IL2RA
CASC5	CMPK2	FBXO39	HAMP	IL4I1
CCL7	CRABP1	FCGR2B	HAPLN3	IRF4
ISG15	MYBL1	PRKCQ	SDS	TMEM229B
ITGA9	NKG7	PTGER2	SH2D1A	TNFSF18
KIAA1199	OR2A5	RGL1	SLA2	TRAT1
KLHDC1	OR5D14	RHBDF2	SLAMF1	TSHZ3
KLRD1	OR6K6	RNASE2	SMOX	TXK
LILRB5	P2RX5	RRM2	SOCS1	TYMS
MERTK	PLEKHO1	RTP4	SSTR2	USP18
MKI67	PRF1	RUNX3	STAB1	
MS4A6E	PRKCA	SAMD3	STAT4	

Table 2Gene Ontology classes enriched in the overall 377 genes (a) and 157 top ranked genes (b) GO categories defined by PANTHER pathway tool [27, 33]

**Table 2a:** 

Analysis Type:	PANTHER Overrepresentation Test (release 20160715)					
**Annotation Version and Release Date:**	**PANTHER version 11.1 Released 2016-10-24**					
**Analyzed List:**	**377genes (Homo sapiens)**					
**Reference List:**	**Homo sapiens (all genes in database)**					
**Bonferroni correction:**	**TRUE**					
**Bonferroni count:**	**158**					
PANTHER Pathways	Homo sapiens - REFLIST (20972)	377genes (385)	377genes (expected)	377genes (over/under)	377 genes (fold Enrichment)	377genes (P-value)
JAK/STAT signaling pathway (P00038)	17	6	0.31	+	19.23	1.50E-04
Toll receptor signaling pathway (P00054)	60	21	1.1	+	19.07	5.00E-18
Interleukin signaling pathway (P00036)	98	23	1.8	+	12.78	4.53E-16
T cell activation (P00053)	96	22	1.76	+	12.48	4.03E-15
B cell activation (P00010)	72	15	1.32	+	11.35	1.83E-09
p38 MAPK pathway (P05918)	42	7	0.77	+	9.08	2.49E-03
Apoptosis signaling pathway (P00006)	122	20	2.24	+	8.93	5.24E-11
Inflammation mediated by chemokine and cytokine signaling pathway (P00031)	261	36	4.79	+	7.51	3.53E-18
Blood coagulation (P00011)	47	6	0.86	+	6.95	4.23E-02
VEGF signaling pathway (P00056)	72	8	1.32	+	6.05	1.08E-02
Ras Pathway (P04393)	76	8	1.4	+	5.73	1.56E-02
CCKR signaling map (P06959)	173	17	3.18	+	5.35	6.02E-06
p53 pathway (P00059)	88	8	1.62	+	4.95	4.19E-02
Angiogenesis (P00005)	176	16	3.23	+	4.95	4.23E-05
Integrin signalling pathway (P00034)	192	17	3.52	+	4.82	2.59E-05
EGF receptor signaling pathway (P00018)	139	11	2.55	+	4.31	1.07E-02
Gonadotropin-releasing hormone receptor pathway (P06664)	235	15	4.31	+	3.48	6.36E-03
Unclassified (UNCLASSIFIED)	18333	232	336.55	-	0.69	0.00E+00

**Table 2b:** 

Analysis Type:	PANTHER Overrepresentation Test (release 20160715)					
**Annotation Version and Release Date:**	**PANTHER version 11.1 Released 2016-10-24**					
**Analyzed List:**	**157 genes (Homo sapiens)**					
**Reference List:**	**Homo sapiens (all genes in database)**					
**Bonferroni correction:**	**TRUE**					
**Bonferroni count:**	**158**					
PANTHER Pathways	Homo sapiens - REFLIST (20972)	157 genes Input (158)	157 genes Input (expected)	157 genes Input (over/under)	157 genes Input (fold Enrichment)	157 genes Input (P-value)
JAK/STAT signaling pathway (P00038)	17	3	0.13	+	23.42	4.94E-02
Interleukin signaling pathway (P00036)	98	9	0.74	+	12.19	1.20E-05
B cell activation (P00010)	72	6	0.54	+	11.06	3.25E-03
Toll receptor signaling pathway (P00054)	60	5	0.45	+	11.06	1.62E-02
T cell activation (P00053)	96	7	0.72	+	9.68	1.55E-03
Inflammation mediated by chemokine and cytokine signaling pathway (P00031)	261	12	1.97	+	6.1	1.34E-04
Integrin signalling pathway (P00034)	192	8	1.45	+	5.53	1.86E-02
Unclassified (UNCLASSIFIED)	18333	96	138.12	-	0.7	0.00E+00

## DISCUSSION

Evolving research from correlative studies as well as clinical trials, including those for UBC, have emphasized the value of the pre-existing tumour immune state as a predictor of response to treatment and survival [29, 30]. Furthermore, UBC is associated with a comparatively high mutational burden [31], which could potentially contribute to increased immunogenicity making them more susceptible to novel immunotherapy-based approaches. In order to gain a better understanding of the pre-existing tumour immune landscape in UBC, we conducted a comprehensive *in silico* immune transcriptomic profiling of the MIBC tumours from the TCGA database.

Four distinct molecular subtypes in MIBC were defined recently based on the TCGA MIBC genome wide profiling datasets [6]. Indeed, variability in subtype nomenclature has been reported [10], which could be attributed to heterogeneity in tissue samples in addition to several other factors such as inclusion of NMIBC cases in classification schemes. However, since the TCGA bladder cohort is enriched for MIBC and gene expression based clusters have been well defined, we specifically used this cohort to address our question on immune gene expression patterns associated with genomic alterations. Based on the distinct immune signature between clusters in cohort 1 (n=122), we were able to assign cohort 2 (n=225) into the associated TCGA clusters using the top 20% of ranked immune genes from the training cohort. Further analysis on the IFN-γ, IFN-α, and cytotoxic genes were then compared in both cohorts. All of these analyses revealed an increased expression of immune-associated genes in Cluster IV and underactive immune environment in Cluster I. Given that specific genetic alterations associate with these molecular subtypes, it seems that anti-tumour immune responses could be partly driven by oncogenic drivers.

Cluster I has been previously reported to show higher expression of *FGFR3* via mutations, amplifications, and other mechanisms [3]. Interestingly this cluster showed a distinct underactive tumour immune state with reduced expression of IFN genes and genes associated with T-helper type-1 response. It is indeed intriguing that tumours with *FGFR3* mutations or overexpression as per previous classification [6], show an increased overall survival, which contradicts the underactive immune state observed here. In contrast, cases in cluster IV showed the most dominant immune response amongst all four clusters. Tumours in this cluster show decreased expression of *PPAR-γ* and *GATA3*, and significantly increased expression of IFN and antigen presentation pathway genes, in addition to MHC class II genes and those involved in T-cell cytolytic activity. Previous reports have shown that based on broader classifications, cases in cluster IV belong to the basal subtype, which shows poor overall survival [8], [9]. One potential contributor to these associations is the increased expression of immune checkpoint molecules such as *CD274 (PD-L1)*, *IDO1* and the immunosuppressive *IL6* in clusters III and IV that potentially lead to increased resistance to cytotoxic killing and poor response to treatment and ultimately poor survival. As previously shown, this cluster also shows higher levels of EMT related genes, indicating more aggressive tumour phenotype [33]. Our recent report demonstrating that higher PD-L1 expression in cancer cells leads to increased drug resistance upon activation by IFN-γ or PD-1 [32] supports this notion. It could thus be speculated that the IFN-γ secreted by activated T-cells, reflected by the increased expression of *GZMA* in these clusters, could be inducing PD-L1 on the cancer cells with further interaction between these leading to T-cell dysfunction. However, the mechanistic basis of these significant associations needs to be explored further. In other cancers such as melanoma, colorectal, and ovarian, higher expression of IFN pathway genes and of those representing an active immune response is associated with a favourable treatment outcome and overall survival. Furthermore, it is also possible that factors other than anti-tumour immune responses contribute to increased survival rates in tumours with *FGFR3* mutations in MIBC.

Increased expression of MHC class II genes *CD74, HLA-DMB,* and *HLA-DQA1* indicate higher tumour antigen processing by the antigen presenting cells in clusters III and IV. This was also confirmed by gene ontology-based analysis, which reflected a dominance of response to IFN-γ, antigen processing and presentation, cytokine mediated signaling, and cell proliferation, NK cell and macrophage activation, and B cell mediated immunity. These enrichments not only confirm the increased active anti-tumour immune response in clusters IV and some of cluster III but also indicate the immunogenic nature of these clusters that could be potentially be driven by higher mutational burden and recognition of immune cells.

Overall, our findings based on comprehensive immune transcriptomic analysis have significant implications in informing treatment decisions based on immune gene expression patterns. Specifically, since immune checkpoint blockade therapy has shown some promise in bladder cancer [15], near future biomarker driven clinical trials will benefit from these findings that emphasize appropriate patient stratification for treatment. Although recent reports have described the presence of T-cell inflamed and non-inflamed MIBC tumours [17, 33], no previous reports have identified associations between immune response and IFN-associated genes with the four molecular MIBC subtypes. Our study is limited by the fact that the TCGA dataset is enriched for MIBC and thus further validations in other cohorts need to be performed; however, these associations are timely and complement the ongoing and future clinical trials based on immune-based therapies. Clinical translation of our findings will most appropriately be addressed by validation of the most significant differentially expressed genes at both transcriptional and proteomic levels in retrospective and prospective pre-treatment bladder tumour specimens. Future investigations by integration of genomic alterations determined by exome and transcriptome sequencing data are key to identifying the genomic determinants of variability in immune response. Finally our study provides an improved understanding of the bladder cancer molecular subtype associated immune gene expression patterns and will significantly impact the design of novel immune based therapies.

## MATERIALS AND METHODS

### Patient tumour samples

The publicly available global transcriptomic sequencing (RNA-Seq) data from 412 MIBC cases, with the corresponding clinical information was downloaded from TCGA data portal (https://gdc-portal.nci.nih.gov/), now part of the National Cancer Institute’s Genetic Data Commons. The cohort was further divided into two cohorts for downstream analysis. For our training cohort (cohort 1) we used data from the previously defined 129 cases from TCGA that were divided into four clusters based on their integrated analysis of mRNA, miRNA, and protein data [6]. Since our objective was to define immune gene expression patterns in treatment naïve tumours, we excluded patients with any previous therapy. Thus a cohort of 122 MIBC cases, divided into four molecular clusters, was used for *in silico* immune profiling. The remaining 283 cases constituted the validation cohort (cohort 2) of which 225 had no prior therapy.

### Design of immune pathway gene panel for *in silico* immune profiling

To investigate the presence of subtype associated immune signatures, we assembled a defined set of 924 immune related genes. This curated list (Table 1) primarily consisted of genes involved in IFN-α (97 genes), IFN-γ (200 genes), and cytotoxic (115 genes) pathways as defined by Gene Set Enrichment Analysis (GSEA) in combination with other immune genes. The nCounter PanCancer Immune profiling panel (722 genes), (http://www.nanostring.com/products/pancancer_immune/) was used to derive the immune response related genes.

### Bioinformatics analysis of RNA-Seq data

We used the upper quartile-normalized RNA-seq data by expectation maximization (RSEM) available for all selected cases at the TCGA data portal. No additional normalization was performed and the expression data were log_2_ transformed. All downstream data analysis was performed in MATLAB (Mathworks, Inc., Natick, Massachusetts, USA). Focusing on the first set of 122 samples, where clustering information is known, we performed separate analyses of the genes in each of the four immune panels (NanoString™, IFN-α, IFN-γ, and T cell cytotoxicity associated genes). Using a feature selection algorithm, genes were ranked based on their ability to discriminate samples belonging to one cluster from the remaining. The feature selection algorithm uses an ensemble of five different machine-learning techniques (unpublished). The analysis resulted in 16 ranking tables, four tables for each immune panel, where each table ranked genes on their ability to discriminate samples in one cluster from the rest.

In the hierarchical clustering analysis, the top 20% of genes in each ranking group were assembled to represent each immune panel, resulting in 377 genes (Nanostring), 44 genes (IFN-α), 91 genes (IFN-γ), and 62 genes (T cell cytotoxicity). A final feature selection ranking was performed where the combined unique set of 924 genes was used. The top 5% of genes in each of the four ranking groups were then merged, resulting in 157 unique genes.

### Gene ontology analysis using PANTHER

We used the Protein Analysis Through Evolutionary Relationships (PANTHER), version 11.0, classification system (http://www.pantherdb.org/) [27] to determine dominant and enriched pathways in the top ranking 377 genes (NanoString panel) that were ranked based on their ability to discriminate samples across the four clusters. We then applied the statistical binomial overrepresentation test, as previously described in PANTHER [27], to derive the most dominant enriched pathways and gene ontology (GO) biological processes in our lists compared to the reference human genome. We performed these tests using both the 377 top ranking NanoString™ genes and 157 top ranked genes from all immune panels combined. The p-values were corrected for multiple testing using Bonferroni correction.

### Validation of immune gene signature

The remaining 298 cases, not included in cohort 1, were treated as a validation group. From this cohort, patients with previous BCG therapy were excluded, leaving 225 cases. In order to assign samples in this set to each of the four clusters, only the top ranked 377 genes from the NanoString panel were used. First, for each cluster, two cluster centroids were computed using the expression data from cohort 1 (n=122; total of 8 cluster centroids). The cluster centroids were computed by taking the mean expression of samples in a given cluster (main cluster) and the mean expression of samples that do not belong to that cluster (alternative cluster). Then, for each sample in cohort 2, the Euclidean distance was computed to each of the 8 cluster centroids. A sample was assigned to a cluster with the smallest distance to the main cluster, but only if the distance to the main cluster was smaller than the distance to the alternative cluster. Alternatively, the sample was assigned to the ‘unclassified’ cluster. Using the newly assigned clustering information and ranked list of genes, unsupervised hierarchical clustering was performed.

## SUPPLEMENTARY MATERIALS TABLES





## References

[1] Canadian Cancer Society’s Advisory Committee on Cancer Statistics (2015). Canadian Cancer Statistics 2015.

[2] Mertens LS, Neuzillet Y, Horenblas S, van Rhijn BW (2014). Landmarks in non-muscle-invasive bladder cancer. Nat Rev Urol.

[3] Choi W, Porten S, Kim S, Willis D, Plimack ER, Hoffman-Censits J, Roth B, Cheng T, Tran M, Lee IL, Melquist J, Bondaruk J, Majewski T (2014). Identification of distinct basal and luminal subtypes of muscle-invasive bladder cancer with different sensitivities to frontline chemotherapy. Cancer Cell.

[4] Mitra AP, Quinn DI, Dorff TB, Skinner EC, Schuckman AK, Miranda G, Gill IS, Daneshmand S (2012). Factors influencing post-recurrence survival in bladder cancer following radical cystectomy. BJU Int.

[5] McConkey DJ, Choi W, Dinney CP (2014). New insights into subtypes of invasive bladder cancer: considerations of the clinician. Eur Urol.

[6] The Cancer Genome Atlas Research Network (2014). Comprehensive molecular characterization of urothelial bladder carcinoma. Nature.

[7] Sjodahl G, Lauss M, Lovgren K, Chebil G, Gudjonsson S, Veerla S, Patschan O, Aine M, Fernö M, Ringnér M, Månsson W, Liedberg F, Lindgren D, Höglund M (2012). A molecular taxonomy for urothelial carcinoma. Clin Cancer Res.

[8] Damrauer JS, Hoadley KA, Chism DD, Fan C, Tiganelli CJ, Wobker SE, Yeh JJ, Milowsky MI, Iyer G, Parker JS, Kim WY (2014). Intrinsic subtypes of high-grade bladder cancer reflect the hallmarks of breast cancer biology. Proc Natl Acad Sci U S A.

[9] Knowles MA, Hurst CD (2015). Molecular biology of bladder cancer: new insights into pathogenesis and clinical diversity. Nat Rev Cancer.

[10] Dadhania V, Zhang M, Zhang L, Bondaruk J, Majewski T, Siefker-Radtke A, Guo C, Dinney C, Cogdell DE, Zhang S, Lee S, Lee JG, Weinstein JN (2016). Meta-analysis of the luminal and basal subtypes of bladder cancer and the identification of signature immunohistochemical markers for clinical use. EBioMedicine.

[11] Kamat AM, Hahn NM, Efstathiou JA, Lerner SP, Malmström PU, Choi W, Guo CC, Lotan Y, Kassouf W (2016). Bladder cancer. Lancet.

[12] McConkey DJ, Choi W, Dinney CP (2015). Genetic subtypes of invasive bladder cancer. Curr Opin Urol.

[13] Kardos J, Chai S, Mose LE, Selitsky SR, Krishnan B, Saito R, Iglesia MD, Milowsky MI, Parker JS, Kim WY, Vincent BG (2016). Claudin-low bladder tumors are immune infiltrated and actively immune suppressed. JCI Insight.

[14] Pardoll DM (2012). The blockade of immune checkpoints in cancer immunotherapy. Nat Rev Cancer.

[15] Rosenberg JE, Hoffman-Censits J, Powles T, van der Heijden MS, Balar AV, Necchi A, Dawsonon N, O'Donnell PH, Balmanoukian A, Loriot Y, Srinivas S, Retz MM, Grivas P (2016). Atezolizumab in patients with locally advanced and metastatic urothelial carcinoma who have progressed following treatment with platinum-based chemotherapy: a single-arm, multicentre, phase 2 trial. Lancet.

[16] El-Osta H, Shahid K, Mills GM, Peddi P (2016). Immune checkpoint inhibitors: the new frontier in non-small-cell lung cancer treatment. Onco Targets Ther.

[17] Sweis RF, Spranger S, Bao R, Paner GP, Stadler WM, Steinberg GD, Gajewski TF (2016). Molecular drivers of the non-T cell-inflamed tumor microenvironment in urothelial bladder cancer. Cancer Immunol Res.

[18] Fridman WH, Pagès F, Sautès-Fridman C, Galon J (2012). The immune contexture in human tumours: impact on clinical outcome. Nat Rev Cancer.

[19] Sharma P, Shen Y, Wen S, Yamada S, Jungbluth AA, Gnjatic S, Bajorin DF, Reuter VE, Herr H, Old LJ, Sato E (2007). CD8 tumor-infiltrating lymphocytes are predictive of survival in muscle-invasive urothelial carcinoma. Proc Natl Acad Sci U S A.

[20] Yuan J, Hegde PS, Clynes R, Foukas PG, Harari A, Kleen TO, Kvistborg P, Maccalli C, Maecker HT, Page DB, Robins H, Song W, Stack EC (2016). Novel technologies and emerging biomarkers for personalized cancer immunotherapy. J Immunother Cancer.

[21] Sjödahl G, Lövgren K, Lauss M, Chebil G, Patschan O, Gudjonsson S, Månsson W, Fernö M, Leandersson K, Lindgren D, Liedberg F, Höglund M (2016). Infiltration of CD3^+^ and CD68^+^ cells in bladder cancer is subtype specific and affects the outcome of patients with muscle-invasive tumors. Urol Oncol.

[22] Zhang Q, Hao C, Cheng G, Wang L, Wang X, Li C, Qiu J, Ding K (2015). High CD4(+) T cell density is associated with poor prognosis in patients with non-muscle-invasive bladder cancer. Int J Clin Exp Pathol.

[23] Meng X, Huang Z, Teng F, Xing L, Yu J (2015). Predictive biomarkers in PD-1/PD-L1 checkpoint blockade immunotherapy. Cancer Treat Rev.

[24] Spranger S, Sivan A, Corrales L, Gajewski TF (2016). Tumor and host factors controlling antitumor immunity and efficacy of cancer immunotherapy. Adv Immunol.

[25] Pitt JM, Vetizou M, Daillere R, Roberti MP, Yamazaki T, Routy B, Lepage P, Boneca IG, Chamaillard M, Kroemer G, Zitvogel L (2016). Resistance mechanisms to immune-checkpoint blockade in cancer: tumor-intrinsic and -extrinsic factors. Immunity.

[26] Au KK, Le Page C, Ren R, Meunier L, Clément I, Tryshkin K, Peterson N, Kendall-Dupont J, Childs T, Francis JA, Graham CH, Craig AW, Squire JA (2016). STAT1-associated intratumoural TH1 immunity predicts chemotherapy resistance in high-grade serous ovarian cancer. J Pathol Clin Res.

[27] Mi H, Muruganujan A, Casagrande JT, Thomas PD (2013). Large-scale gene function analysis with the PANTHER classification system. Nat Protoc.

[28] Weinstein JN, Kim J, Creighton CJ, Akbani R, Hoadley KA, Kim WY, Morgan MB, Hinoue T, Cherniack A, Su X, Mungall AJ, Ryan MC, Bajorin DF (2015). Progress in The Cancer Genome Atlas bladder cancer project. Cancer Res.

[29] Santarpia M, Karachaliou N (2015). Tumor immune microenvironment characterization and response to anti-PD-1 therapy. Cancer Biol Med.

[30] Farkona S, Diamandis EP, Blasutig IM (2016). Cancer immunotherapy: the beginning of the end of cancer?. BMC Med.

[31] Cazier JB, Rao SR, McLean CM, Walker AK, Wright BJ, Jaeger EE, Kartsonaki C, Marsden L, Yau C, Camps C, Kaisaki P, Taylor J, Catto JW (2014). Oxford-Illumina WGS500 Consortium. Whole-genome sequencing of bladder cancers reveals somatic CDKN1A mutations and clinicopathological associations with mutation burden. Nat Commun.

[32] Black M, Barsoum IB, Truesdell P, Cotechini T, Macdonald-Goodfellow SK, Petroff M, Siemens DR, Koti M, Craig AW, Graham CH (2016). Activation of the PD-1/PD-L1 immune checkpoint confers tumor cell chemoresistance associated with increased metastasis. Oncotarget.

[33] Seiler R, Hussam Ashab HA, Erho N, van Rhijn BW, Winters B, Douglas J, Van Kessel KE, Fransen van de Putte EE, Sommerlad M, Wang NQ, Choeurng V, Gibb EA, Palmer-Aronsten B (2017). Impact of molecular subtypes in muscle-invasive bladder cancer on predicting response and survival after neoadjuvant chemotherapy. Eur Urol.

