# Supplementary material for: Comprehensive immune transcriptomic analysis in bladder cancer reveals subtype specific immune gene expression patterns of prognostic relevance
Source: Oncotarget. 2017 Aug 9;8(41):70982–1001. doi: 10.18632/oncotarget.20237 (PMC5642612; doi:10.18632/oncotarget.20237)
Supplement: Supplementary file 1 [file oncotarget-08-70982-s001.pdf]

Comprehensive immune transcriptomic analysis in bladder cancer reveals subtype specific immune gene expression patterns of prognostic relevance

SUPPLEMENTARY MATERIALS

Supplementary Table 1: Top 20% of NanoString nCounter PanCancer immune panel genes (n=377) after feature selection

See Supplementary File 1

Supplementary Table 2: Top 5% of genes derived from all 4 immune panels (n=157) after feature selection

A2M	CD44	FCGR2A	JAK3	SELP
ADA	CD48	FCGR3A	JAM3	SELPLG
ADAM19	CD53	FN1	LATS2	SERPINB2
ADORA3	CD63	FOXJ1	LCP2	SERPING1
AKT3	CD79A	GAS6	LILRB1	SIGIRR
ANXA1	CD86	GATA3	LILRB2	SSTR2
AXL	CD8B	GBP6	LY96	ST3GAL5
BANK1	CDK6	GIMAP4	MSR1	STAT3
BATF	CEACAM6	GIMAP5	MT2A	THBD
BCL10	CFD	HAVCR2	MYBL1	THBS1
BCL2L1	CH25H	HS3ST3B1	MYD88	THY1
BPGM	CMKLR1	HREF	NCAM1	TLR1
BTK	COL3A1	ICAM1	NOD2	TLR4
C1QA	COLEC12	IFI16	NOTCH1	TNFAIP2
C1QB	CREB5	IFI30	NT5E	TNFAIP6
C1R	CSF1	IFITM3	PBK	TNFRSF13B
C1S	CSF1R	IL12RB1	PDCD1	TNFRSF17
C2	CSF2RB	IL12RB2	PDGFC	TNFRSF18
C3AR1	CSF3	IL15	PDGFRB	TNFRSF8
CARD11	CTSH	IL1R1	PIK3CD	TNFSF12
CASP1	CTSW	IL1RAP	PLEKHO1	TNFSF13B
CCL15	CXCL1	IL2RA	PPARG	TNFSF4
CCL19	CXCR3	IL4I1	PTGER2	TPSAB1
CCL8	CYBB	IL5RA	PTPN6	TRAF3
CCR1	CYFIP2	ILF3	PTPRC	TRIM14
CCR2	EGR2	IRF5	RRM2	TXK
CCR5	EOMES	IRF8	RUNX3	VCAM1
CD14	F12	ITGA5	S100A12	WARS
CD200	FAM20A	ITGA6	S100A7	ZAP70
CD22	FAM26F	ITGAM	S100A8	
CD28	FAM40B	ITGAX	SAMD9	
CD33	FCGR1A	ITGB2	SELL	

Supplementary Table 3: List of eanriched GO biological processes defined by PANTHER analysis [27] in the 377 differentially expressed gene list

See Supplementary File 1

Supplementary Table 4: List of enriched GO biological processes defined by PANTHER analysis [27] in the top ranked 157 genes

See Supplementary File 1
