# Supplementary material for: Comprehensive immune transcriptomic analysis in bladder cancer reveals subtype specific immune gene expression patterns of prognostic relevance
Source: Oncotarget. 2017 Aug 9;8(41):70982–1001. doi: 10.18632/oncotarget.20237 (PMC5642612; doi:10.18632/oncotarget.20237)
Supplement: Supplementary file 2 [file oncotarget-08-70982-s002.docx]

| Supplementary Table 1. Top 20% of NanoString nCounter PanCancer immune panel genes (n=377) after feature selection | | | | |
| --- | --- | --- | --- | --- |
| A2M  ABCB1  ADA  ADORA2A  AKT3  ANP32B  ANXA1  APOE  ARG2  ATF1  AXL  BATF  BCL2  BCL2L1  BID  BIRC5  BLK  BST1  BTK  BTLA  C1QA  C1QB  C1QBP  C1R  C1S  C2  C3AR1  C6  C7  C8A  CARD11  CARD9  CASP1  CCL11  CCL14  CCL15  CCL16  CCL19  CCL20  CCL21  CCL25  CCL26  CCL4  CCL5  CCL8 | CCR1  CCR2  CCR5  CCR7  CD14  CD160  CD163  CD180  CD19  CD1D  CD2  CD200  CD209  CD22  CD24  CD247  CD27  CD274  CD28  CD33  CD34  CD37  CD38  CD3D  CD3E  CD4  CD44  CD46  CD47  CD48  CD5  CD53  CD55  CD58  CD59  CD6  CD63  CD68  CD7  CD74  CD79A  CD79B  CD80  CD84  CD86 | CD8A  CD9  CD96  CD99  CDH1  CDH5  CDK1  CDKN1A  CEACAM6  CEACAM8  CFD  CFP  CHUK  CKLF  CLEC4A  CLEC5A  CLU  CMA1  CMKLR1  COL3A1  COLEC12  CREB5  CSF1  CSF1R  CSF2RB  CSF3  CTSG  CTSH  CTSL1  CTSS  CTSW  CX3CL1  CXCL1  CXCL12  CXCL2  CXCL9  CXCR3  CXCR5  CXCR6  CYBB  CYFIP2  CYLD  DEFB1  DPP4  DUSP6 | EGR2  ELANE  ENTPD1  EOMES  ETS1  F13A1  F2RL1  FADD  FAS  FCER1G  FCER2  FCGR1A  FCGR2A  FCGR2B  FCGR3A  FN1  FOXJ1  FUT7  FYN  GATA3  GNLY  GPI  GPR44  GZMB  GZMK  GZMM  HAVCR2  HCK  HLA-DMB  HLA-DPA1  HLA-DPB1  HLA-DQA1  HLA-DQB1  HLA-DRA  HRAS  HSD11B1  ICAM1  ICAM2  ICAM3  ICOSLG  IDO1  IFI16  IFI27  IFITM1  IFNA17 | IFNA2  IFNAR2  IFNGR1  IGF1R  IGF2R  IL10  IL10RA  IL12RB1  IL12RB2  IL13RA1  IL15  IL15RA  IL16  IL17F  IL17RA  IL17RB  IL18  IL19  IL1B  IL1R1  IL1R2  IL1RAP  IL1RAPL2  IL1RL2  IL1RN  IL21R  IL22  IL22RA1  IL22RA2  IL24  IL27  IL2RA  IL2RB  IL2RG  IL32  IL34  IL4R  IL5RA  IL6R  IL7R  ILF3  IRAK1  IRAK2  IRF4  IRF5 |
| Supplementary Table 1 cont. | | | | |
| IRF8  ISG20  ITGA2  ITGA4  ITGA5  ITGA6  ITGAL  ITGAM  ITGAX  ITGB2  JAK3  JAM3  KIT  KLRK1  LAG3  LAMP2  LCK  LCN2  LCP1  LGALS3  LILRA1  LILRA5  LILRB1  LILRB2  LILRB3  LRP1  LTA  LTB  LTF  LY86  LY9 | LY96  MAF  MAP2K1  MAP2K4  MAP3K5  MAP3K7  MAPK14  MARCO  MASP1  MEF2C  MEFV  MERTK  MFGE8  MICB  MRC1  MS4A1  MS4A2  MSR1  MYD88  NCAM1  NCR1  NFATC1  NFATC4  NLRP3  NOD2  NOTCH1  NT5E  NUP107  PBK  PDCD1  PDCD1LG2 | PDGFC  PDGFRB  PIK3CD  PLA2G1B  PLAU  PLAUR  PNMA1  POU2F2  PPARG  PRAME  PRF1  PRKCD  PRKCE  PSMB7  PSMB8  PSMB9  PTPRC  RELB  REPS1  RRAD  RUNX3  S100A12  S100A7  S100A8  SAA1  SBNO2  SELL  SELPLG  SERPINB2  SERPING1  SH2B2 | SIGIRR  SIGLEC1  SLAMF1  SLAMF7  SLC11A1  SMAD3  SMPD3  SOCS1  SPINK5  SPN  SPO11  SPP1  STAT3  STAT4  SYT17  TAP1  TAP2  TBK1  TBX21  TCF7  THBD  THBS1  THY1  TIRAP  TLR1  TLR2  TLR4  TLR6  TLR7  TLR8  TLR9 | TMEFF2  TNFRSF13B  TNFRSF14  TNFRSF17  TNFRSF18  TNFRSF1B  TNFRSF4  TNFRSF8  TNFRSF9  TNFSF10  TNFSF12  TNFSF13B  TNFSF14  TNFSF4  TOLLIP  TPSAB1  TPTE  TRAF2  TRAF3  TREM1  TREM2  TTK  TXK  TXNIP  TYK2  VCAM1  VEGFA  ZAP70 |

Supplementary Table 3

| Analysis Type: | PANTHER Overrepresentation Test (release 20160715) | | | | | |
| --- | --- | --- | --- | --- | --- | --- |
| Annotation Version and Release Date: | PANTHER version 11.1 Released 2016-10-24 | | | |  |  |
| Analyzed List: | 377genes (Homo sapiens) | |  |  |  |  |
| Reference List: | Homo sapiens (all genes in database) | | |  |  |  |
| Bonferroni correction: | TRUE |  |  |  |  |  |
| Bonferroni count: | 241 |  |  |  |  |  |
| PANTHER GO-Slim Biological Process | Homo sapiens - REFLIST (20972) | 377genes (385) | 377genes (expected) | 377genes (over/under) | 377genes (fold Enrichment) | 377genes (P-value) |
| response to interferon-gamma (GO:0034341) | 59 | 17 | 1.08 | + | 15.7 | 6.92E-13 |
| antigen processing and presentation of peptide or polysaccharide antigen via MHC class II (GO:0002504) | 40 | 11 | 0.73 | + | 14.98 | 9.11E-08 |
| hemopoiesis (GO:0030097) | 95 | 23 | 1.74 | + | 13.19 | 3.56E-16 |
| cytokine-mediated signaling pathway (GO:0019221) | 175 | 41 | 3.21 | + | 12.76 | 2.88E-29 |
| localization (GO:0051179) | 123 | 25 | 2.26 | + | 11.07 | 6.40E-16 |
| cell proliferation (GO:0008283) | 144 | 28 | 2.64 | + | 10.59 | 1.79E-17 |
| locomotion (GO:0040011) | 156 | 29 | 2.86 | + | 10.13 | 1.27E-17 |
| cellular defense response (GO:0006968) | 208 | 35 | 3.82 | + | 9.17 | 3.72E-20 |
| natural killer cell activation (GO:0030101) | 69 | 10 | 1.27 | + | 7.89 | 2.05E-04 |
| blood coagulation (GO:0007596) | 91 | 12 | 1.67 | + | 7.18 | 4.51E-05 |
| immune response (GO:0006955) | 375 | 45 | 6.88 | + | 6.54 | 1.75E-20 |
| MAPK cascade (GO:0000165) | 256 | 28 | 4.7 | + | 5.96 | 2.79E-11 |
| macrophage activation (GO:0042116) | 119 | 12 | 2.18 | + | 5.49 | 7.14E-04 |
| response to external stimulus (GO:0009605) | 220 | 22 | 4.04 | + | 5.45 | 6.60E-08 |
| regulation of catalytic activity (GO:0050790) | 213 | 20 | 3.91 | + | 5.11 | 1.22E-06 |
| B cell mediated immunity (GO:0019724) | 214 | 18 | 3.93 | + | 4.58 | 3.61E-05 |
| complement activation (GO:0006956) | 131 | 10 | 2.4 | + | 4.16 | 4.61E-02 |
| cell-cell adhesion (GO:0016337) | 305 | 22 | 5.6 | + | 3.93 | 2.13E-05 |
| cell adhesion (GO:0007155) | 154 | 11 | 2.83 | + | 3.89 | 3.95E-02 |
| cell-cell signaling (GO:0007267) | 156 | 11 | 2.86 | + | 3.84 | 4.41E-02 |
| cellular component movement (GO:0006928) | 413 | 29 | 7.58 | + | 3.82 | 3.32E-07 |
| response to biotic stimulus (GO:0009607) | 175 | 12 | 3.21 | + | 3.74 | 2.94E-02 |
| single-multicellular organism process (GO:0044707) | 363 | 24 | 6.66 | + | 3.6 | 2.69E-05 |
| transmembrane receptor protein tyrosine kinase signaling pathway (GO:0007169) | 200 | 13 | 3.67 | + | 3.54 | 2.62E-02 |
| response to stress (GO:0006950) | 860 | 53 | 15.79 | + | 3.36 | 5.32E-12 |
| cell differentiation (GO:0030154) | 442 | 27 | 8.11 | + | 3.33 | 2.07E-05 |
| regulation of phosphate metabolic process (GO:0019220) | 379 | 22 | 6.96 | + | 3.16 | 7.43E-04 |
| apoptotic process (GO:0006915) | 330 | 18 | 6.06 | + | 2.97 | 1.29E-02 |
| response to stimulus (GO:0050896) | 1175 | 45 | 21.57 | + | 2.09 | 7.88E-04 |
| regulation of biological process (GO:0050789) | 1613 | 56 | 29.61 | + | 1.89 | 8.86E-04 |
| Unclassified (UNCLASSIFIED) | 8633 | 95 | 158.48 | - | 0.6 | 0.00E+00 |

Supplementary Table 4

| Analysis Type: | PANTHER Overrepresentation Test (release 20160715) | | | | |  |
| --- | --- | --- | --- | --- | --- | --- |
| Annotation Version and Release Date: | PANTHER version 11.1 Released 2016-10-24 | | | |  |  |
| Analyzed List: | 157 genes (Homo sapiens) | | |  |  |  |
| Reference List: | Homo sapiens (all genes in database) | | |  |  |  |
| Bonferroni correction: | TRUE |  |  |  |  |  |
| Bonferroni count: | 241 |  |  |  |  |  |
| PANTHER GO-Slim Biological Process | Homo sapiens - REFLIST (20972) | Analyzed genes (from 157 top ranked genes) | 157 genes Input (expected) | 157 genes Input (over/under) | fold Enrichment from analyzed gene set (157 top ranked genes) | 157 genes (P-value) |
| response to interferon-gamma (GO:0034341) | 59 | 7 | 0.44 | + | 15.75 | 9.89E-05 |
| hemopoiesis (GO:0030097) | 95 | 8 | 0.72 | + | 11.18 | 1.88E-04 |
| cell proliferation (GO:0008283) | 144 | 11 | 1.08 | + | 10.14 | 4.11E-06 |
| macrophage activation (GO:0042116) | 119 | 9 | 0.9 | + | 10.04 | 9.22E-05 |
| locomotion (GO:0040011) | 156 | 9 | 1.18 | + | 7.66 | 8.33E-04 |
| localization (GO:0051179) | 123 | 7 | 0.93 | + | 7.55 | 1.13E-02 |
| cytokine-mediated signaling pathway (GO:0019221) | 175 | 9 | 1.32 | + | 6.83 | 2.08E-03 |
| immune response (GO:0006955) | 375 | 16 | 2.83 | + | 5.66 | 7.85E-06 |
| Unclassified (UNCLASSIFIED) | 8633 | 44 | 65.04 | - | 0.68 | 0.00E+00 |
